# Patent pooling to increase access to essential medicines

**DOI:** 10.2471/BLT.18.229179

**Published:** 2019-06-11

**Authors:** Esteban Burrone, Dzintars Gotham, Andy Gray, Kees de Joncheere, Nicola Magrini, Yehoda M Martei, Charles Gore, Marie Paule Kieny

**Affiliations:** a Medicines Patent Pool, 7 Rue de Varembé, 1202 Geneva, Switzerland.; b Division of Pharmacology, University of KwaZulu-Natal, Durban, South Africa.; c Netherlands Antibiotic Development Platform, University Medical Centre, Utrecht, Netherlands.; d Essential Medicines and Health Products Department, World Health Organization, Geneva, Switzerland.; e Hematology-Oncology Division, University of Pennsylvania, Philadelphia, United States of America.

Access to medicines is key to achieving universal health coverage (UHC); however, such access can be hindered by unaffordable prices. A good example of improvement in access to medicines is treatment for human immunodeficiency virus (HIV) infection. Global coverage was very low in 2000, with only 611 000 people receiving treatment; however, in 2017, 21.7 million people were on treatment.^1^^,^^2^ This increase was partly due to access to affordable, quality-assured generic HIV medicines in low- and middle-income countries.^1^

One way to achieve better access to new medicines is patent pools, which allow third parties to acquire non-exclusive licences for the intellectual property needed to develop products. While patent pools have existed for several decades in other fields of technology, such as in digital technologies, they are a relatively new concept in public health, where they have been applied to address some of the access challenges in low- and middle-income countries.^3^

To improve access to antiretroviral treatment in low- and middle-income countries, Unitaid established the Medicines Patent Pool in 2010 as the first public health patent pool. Later, the patent pool’s mandate was expanded to treatments for tuberculosis and hepatitis C. The patent pool has negotiated most of the licensing agreements with pharmaceutical companies that have enabled competitive generic manufacture of antiretrovirals in low- and middle-income countries before patent expiry. These licences have also facilitated the development of new formulations that are particularly needed in resource-constrained settings, such as certain fixed-dose combinations and paediatric formulations. To date, the patent pool’s generic partners have distributed 22 million patient-years of treatment, allowing global savings of 1.06 billion United States dollars (US$), according to a biannual analysis undertaken by a leading auditing company.^4^

In 2016, the *Lancet* Commission on Essential Medicines Policies, the World Health Organization (WHO) and other stakeholders called for the patent pool to expand its mandate to a broader range of patented essential medicines.^5^^,^^6^ Here, we outline the findings of a released feasibility study on expanding the patent pool’s mandate,^7^ laying out the public health case for adapting its model to disease areas beyond the initial three focus diseases. In May 2018, the patent pool acted on the results of the feasibility study and expanded its mandate to include other patented essential medicines.

## Essential medicines

WHO’s *Model list of essential medicines* comprises medicines that meet the priority health needs of the population and that should always be available and affordable in all countries. WHO’s model list is updated every two years by the Expert Committee on the Selection and Use of Essential Medicines, which considers submissions based on public health relevance, efficacy, safety and comparative cost–effectiveness.^8^

Competitive generic manufacture could lead to significant price reductions for patented essential medicines in low- and middle-income countries. Such reductions have recently taken place for certain oncology medicines following patent expiry and generic market entry. For example, in South Africa, the price of a leukaemia medicine on WHO’s model list has decreased by 98.5% since 2012, to US$ 400 per patient per year in 2019, following generic market entry.^9^^,^^10^ Therefore, public-health-oriented licensing could contribute to price reductions for other essential medicines that are still under patent protection in low- and middle-income countries.

The feasibility study identified various categories of patented medicines for which public health-oriented licensing could be an important strategy to improve access (Table 1). These categories include patented medicines on WHO’s model list and others that have received favourable clinical assessments by the expert committee. We analysed relevant epidemiology, treatment landscapes, market sizes and pricing in low- and middle-income countries, supplemented with case studies from several countries to better understand local contexts.^7^ As patents are granted at the national, or regional level, and patent status (that is, whether a drug is protected by patents) can vary significantly between countries, we also undertook an analysis of the patent landscape in a sample of low- and middle-income countries.^7^ Finally, we looked at the potential impact of the patent pool’s expansion, the possible challenges in ensuring that licences result in increased access and possible ways of addressing such challenges.

**Table 1:** Patented essential medicines for which public health-oriented licensing could be an important strategy to improve access

Category	Description	Case study
Patented medicines in WHO’s model list of essential medicines	The Medicines Patent Pool already works in HIV, tuberculosis and hepatitis C, for which several patented medicines are on WHO’s model list. Outside of these areas, several the medicines on WHO’s model list are under patent protection. Two recent examples are the leukaemia treatments dasatinib and nilotinib, added in 2017.	About 181 000 people in low- and middle-income countries have Philadelphia chromosome positive chronic myeloid leukaemia or acute lymphoblastic leukaemia and could benefit from long-term access to dasatinib and nilotinib. Access gaps remain in several of these countries despite donation or discount programmes.
Patented medicines that are not yet included in WHO’s model list, in part due to insufficient cost–effectiveness at current prices	These are medicines for which the expert committee noted clinical benefits, but had concerns about affordability, particularly in low- and middle-income countries. Examples include novel oral anticoagulants, which offer certain advantages over the previous standard of care (warfarin), including lower monitoring requirements.	An estimated 9.3 million people could benefit from access to novel oral anticoagulants in low- and middle-income countries. Access is currently limited, with vitamin K antagonists remaining the standard of care despite significant practical challenges in using them. Access to these drugs could increase the number of people on anticoagulation with a reduction in cases of venous thromboembolism, stroke or systemic embolism.
Patented medicines that show promising clinical data and may merit inclusion in WHO’s model list in the future if clinical benefit is confirmed	These are medicines with promising clinical data, but for which additional data would be needed to confirm findings. Examples include new oral medicines for the treatment of type 2 diabetes, the SGLT2 inhibitors, which decrease cardiovascular mortality and are reno-protective.	An estimated 93 million people in low- and middle-income countries have type 2 diabetes that is not controlled by metformin alone and may therefore benefit from access to newer oral medicines, reducing the number of major cardiovascular events among people with the disease.
Patented cancer medicines that will be considered at the next WHO model list expert committee review	WHO has established a working group to assess the magnitude of therapeutic effect that would be required for cancer medicines to be considered candidates for addition to WHO’s model list.	Examples include patented medicines for breast cancer (e.g. pertuzumab, trastuzumab emtansine), lung cancer (e.g. afatinib), prostate cancer (e.g. abiraterone, enzalutamide), and multiple myeloma (e.g. lenalidomide, bortezomib). Several of these medicines offer survival benefits and/or improved quality of life.
New antimicrobials	Numerous stakeholders working in antimicrobial resistance have suggested that the patent pool could play an important role in supporting the triple goals of enabling affordable access to novel antimicrobials, facilitating innovation and supporting good stewardship practices.^11^^,^^12^	In 2019, seven new patented antibiotics were submitted for inclusion in WHO’s model list in the context of WHO’s new Access, Watch and Reserve categories. The Medicines Patent Pool licences tailored to the antimicrobial resistance context could offer a mechanism for contributing to achieve access and stewardship goals in low- and middle-income countries.

HIV: Human immunodeficiency virus; SGLT2: sodium-glucose transport protein 2; WHO: World Health Organization.

Based on assessments made by WHO’s expert committee, the feasibility study identified five categories of products that could be potential candidates for licensing via the patent pool. These categories are: (i) patented medicines included in WHO’s model list; (ii) patented medicines that are not yet included in the list, in part due to insufficient cost–effectiveness at current prices; (iii) patented medicines that show promising clinical data and may merit inclusion in the list in the future if clinical benefit is confirmed; (iv) patented cancer medicines that require further review; and (v) new patented antimicrobials (Table 1).

The patent pool’s current focus is primarily on small-molecule medicines, rather than biotherapeutics, as further analysis is required to better understand the potential for patent pooling to contribute to affordable access to biotherapeutics.

## Early licensing

The patent pool’s model contributes to accelerating affordable access to new, improved treatments in low- and middle-income countries in partnership with the pharmaceutical industry. To achieve this goal in HIV, licensing arrangements for new medicines considered promising by WHO has sometimes been necessary before their inclusion in WHO’s model list, as in the case of dolutegravir. The patent pool signed a licensing agreement with a pharmaceutical company for dolutegravir in 2014. By the time the medicine was added to WHO’s model list in 2017, several patent pool sub-licensees had filed for approval of generic versions, including a new fixed-dose combination, with the WHO prequalification programme.

Under an expanded mandate, the patent pool will work in partnership with WHO and other key stakeholders to identify promising medicines for which agreements with patent-holders can be established early, to accelerate access to important new treatments in low- and middle-income countries.

## Challenges and opportunities

As the patent pool begins work in new disease areas, several challenges could arise, such as constrained health systems and limited diagnostic capacity in many low- and middle-income countries, particularly in relation to certain cancers. Another challenge is the lack of large, international donors for scaling up treatments and contributing to the introduction of new products outside the domains of HIV, tuberculosis and malaria. This challenge has already been encountered in hepatitis C, where uptake of products licensed by the patent pool was initially limited to the private market due to the lack of large viral hepatitis treatment programmes. However, more recently, government treatment programmes have begun to be established in certain low- and middle-income countries in the context of broader national strategies on viral hepatitis, partly thanks to access to more affordable medicines.

Governments and global health actors are increasingly recognizing the need for expanded treatment programmes for noncommunicable diseases. Political commitments at the recent United Nations high-level meeting on noncommunicable diseases will need to be followed by robust action to attain UHC. Access to affordable treatments can contribute to the development of public health programmes, including for more active screening and diagnosis. Greater availability and affordability of improved treatments with greater efficacy, easier administration, lesser side-effects or less monitoring requirements can also contribute to reducing health systems costs (Table 1).

The work of the patent pool takes place in the wider context of various policies, instruments and initiatives that can contribute to addressing the challenges in making essential medicines available, affordable and accessible in resource-constrained settings. Patent pooling is one way of contributing to the affordability of products and over the years, governments have taken different approaches to ensuring affordable access to medicines. Approaches have included price negotiations, health technology assessments, reference pricing, new procurement models such as a subscription model and the use of flexibilities under the *Agreement on Trade-Related Aspects of Intellectual Property Rights*. For patent pooling to succeed in new areas, the patent pool will need to work closely with various partners, particularly governments, to explore other barriers to access and to facilitate uptake of products licensed by the patent pool.

Public health-oriented patent pooling has become a proven access strategy since the establishment of the patent pool. In our feasibility analysis, we found that licensing through the patent pool could offer significant public health impact. In many low- and middle-income countries, some of the newer medicines for noncommunicable diseases are not available, not registered or used only by a small proportion of the population, mostly in the private market, with significant out-of-pocket payments. Licensing through the patent pool could potentially accelerate access to improved treatments in countries that might not be considered as significant commercial markets. Suitable royalty provisions could provide adequate compensation to patent holders, while enabling access to lower-priced alternatives.

As a next step, the patent pool is working closely with WHO and other key stakeholders to develop a robust framework for identifying priority medicines where a patent pooling model could have the greatest public health impact and enable wider access to essential medicines in low- and middle-income countries.
